# “Characterization of Nanoparticles Intended for Drug Delivery”

**DOI:** 10.3797/scipharm.br-11-01

**Published:** 2011-09-30

**Authors:** Franz Gabor

**Affiliations:** Department of Pharmaceutical Technology & Biopharmaceutics, University of Vienna, Austria

Not least because of the nano-hype, a myriad of labs focuses their research on nanoparticles. Inexplicably, a collection of lab protocols was not available until now. That way, this book fills a gap by offering a basic set of methods for pre-clinical characterization of nanomaterials provided by experienced people from the National Cancer Institute’s Nanotechnology Lab.

Before starting the experimental work, reading chapter 2 dealing with the challenges of nanoparticle characterization is highly recommended. Due to the unique properties of nanoparticles, interference with standardized protocols can lead to false-positive or false-negative results. Being aware of these problems the assay design as well as the interpretation of results might be adapted accordingly.

Each chapter starts with a short description of the basics, and after listing the materials necessary for the assay, mostly a detailed description of the protocol should allow for a successful experiment. Most useful, the section “notes” gives valuable hints towards pitfalls, selection of devices, data analysis, and interpretation of results. Finally, for a more detailed knowledge, a short list of references rounds off each chapter.

As virtually impossible, this book offers not a complete, but a highly comprehensive and broad spectrum of protocols. The toolset provided starts with methods for physicochemical characterization of nanoparticles such as size, zeta potential, imaging methods (SEM, TEM, AFM) and molecular weight of dendrimers. To account for sterility, two versions of the limulus test as well as a general test for microbial contamination are given. The topic “in-vitro detection and quantitation” lists only methods for gold and fullerene nanoparticles, and should be extended to pharmaceutically relevant polymers in future. The often controversy discussed issue “cytotoxicity” is covered by a broad spectrum of different approaches: Estimation of cell-viability by MTT- or LDH-assay, apoptotic potential by caspase activation assay, oxidative stress by even three different protocols comprising reactive oxygen production, lipid peroxidation and glutathione/oxidized glutathione determination as well as effects on lysosomes such as accumulation of autophagic vacuoles and total lysosome content. Finally, some light is shed on the influence of nanomaterials on blood constituents ranging from hemolysis, thrombogenicity and complement activation to chemotaxis. For the next edition of this high quality collection of protocols it would be desirable to include also techniques for elucidation of bioadhesivity, cell-binding, uptake and transcellular transport as required for ex-vivo characterization of drug-loaded nanoparticles.

All in all, this collection of lab-protocols is well recommended for scientists, starters in the field but also people dealing with regulatory issues in the field of nanomedicine.

